# Proteomic Analysis of Fibroblasts Exposed to Resin Composite Release

**DOI:** 10.1002/prca.202400049

**Published:** 2024-10-18

**Authors:** Yohann Flottes, Elisabeth Dursun

**Affiliations:** ^1^ URP 4462 Innovative Dental Materials and Interfaces Research Unit Paris Cité University Montrouge France; ^2^ Henri Mondor Hospital, AP‐HP Créteil France; ^3^ Department of Prosthodontics Faculty of Dental Surgery Paris Cité University Montrouge France; ^4^ Department of Pediatric Dentistry Faculty of Dental Surgery Paris Cité University Montrouge France

**Keywords:** adverse effects, composite resins, dental materials, methylmethacrylate, proteomics, toxicity

## Abstract

**Objective:**

To investigate the potential effects of products released by a resin composite on the proteome of human gingival fibroblasts.

**Methods:**

Fifteen resin composite cylinders of a Bis‐GMA‐based resin composite (Tetric EvoCeram, Ivoclar) were made and placed in a culture medium for 24 h. Then, 30 mL of this medium was placed for 72 h in contact with human gingival fibroblasts and a second control group consisted of cells placed in culture medium only. Afterward, cells were collected, washed, and their proteins extracted. Three two‐dimensional electrophoresis were performed per condition. Image analysis of the gels was carried out to highlight the differential protein spots. These spots were then analyzed by an ESI/qTOF mass spectrometer. Finally, specific databases provided protein identification, their interactions, and the pathways where they are implicated.

**Results:**

Delta2D software allowed the detection of 21 spots of different proteins. The MASCOT identified 28 proteins. Five proteins from four spots were upregulated, 23 proteins from 17 spots were downregulated. The UniProt database showed that all these proteins were involved in cellular architecture, structural modifications and quality control of proteins, cellular homeostasis, and metabolic pathways. The STRING database revealed the interactions between the regulated proteins. The GO enrichment analysis showed that 19 pathways were affected.

**Significance:**

The products released from the resin composite tested led to changes in the fibroblast proteome. Under the conditions of this study, resin composite released products can cause early adverse effects on cells, but without complete inhibition of their cellular functions.

## 1 Introduction

Dental resin composites are widely used in clinical practice due to their high strength, resistance, and aesthetic properties for treating small‐ and medium‐sized carious lesions [1]. In 2014, more than 1.1 billion dental restorations were placed worldwide, of which 800 million were direct resin composite restorations [2]. These resins consist of inorganic fillers, an organic matrix, and a coupling agent. The resin matrix typically includes methacrylate monomers, such as Bis‐GMA, UDMA, Bis‐EMA, TEGDMA, or HEMA [3]. Despite their mechanical and aesthetic advantages, resin composites have limitations due to their chemical composition and setting reaction, including incomplete polymerization, relatively low resistance to wear, polymerization shrinkage, and microleakage. Additionally, degradation over time, which results from wear or chemical degradation, remains a concern. In fact, incomplete polymerization and degradation can lead to the release of products that may elicit local and systemic body reactions [4]. The release of monomers into surrounding tissues implies risks of adverse effects at the gingival margins and the dentin‐pulp complex, depending on the restorative location, the amount, and the type of monomers released [5, 6]. Moreover, some released products can react with nucleophilic centers of proteins, lipids, and/or DNA, potentially resulting in the production of cytotoxic and/or genotoxic substances [7]. Numerous studies and systematic reviews have pointed out the potential exposure to bisphenol A (BPA), particularly in resin composites containing BPA‐derived monomers, that is, most of them [8].

Many studies have reported the cytotoxic effects of these monomers, typically using the dimethylthiazol‐2‐yl diphenyltetrazolium bromide (MTT) assay [9] or by analyzing the formation of reactive oxygen species (ROS) [10]. More detailed information is needed to understand the cellular mechanisms behind this cytotoxicity, including cellular adaptation, through the analysis of proteome changes following initial interactions with the products released by resin composites.

As such, proteomics consists in the analysis of the entire protein content expressed in a genome (i.e., the proteome), at a given time. Its analysis may provide valuable information about molecular mechanisms governing homeostatic cell state and responses to external disturbances, including data on protein location, up‐ and downregulation, and, post‐translational modifications (PTMs). Such proteome modifications can be used to assess cellular reactions to specific substances, helping to evaluate their toxicity [11, 12, 13, 14]. To our knowledge, no studies have explored the effects of monomer release from resin composite on cellular metabolism in a context similar to clinical conditions.

The objective of this study was to investigate the potential effects of products released from a resin composite on the proteome of gingival fibroblasts.

## 2 Methods

**FIGURE 1:**
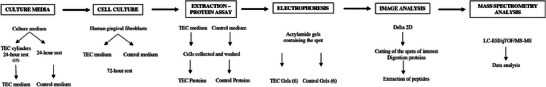
Summary of the experiment.

A summary of the experiments is presented in Figure 1.

### 2.1 Materials, Cells, Culture Medium, and Chemicals

The resin composite Tetric Evo‐Ceram (TEC, Ivoclar, Schaan, Liechtenstein) was selected. Its MSDS sheet indicates that it consists of 5%–10% urethane dimethacrylate (UDMA), 5%–7% bisphenol A‐glycidyl methacrylate (bis‐GMA), and 3%–5% bisphenol A‐ethoxylate dimethacrylate (Bis‐EMA). Cylinders (3 mm base diameter, 4 mm height) were produced using Teflon molds. A first 2 mm increment was inserted into the mold and light‐cured for 20 s (VALO, Ultradent, South Jordan, UT, USA, standard power 1000 mW/cm^2^), then a second 2 mm increment was added and also light‐cured for 20 s.

Human gingival fibroblasts from primary cell culture were used.

The culture medium was prepared from Dulbecco's Modified Eagle Medium (DMEM) (1X) consisting of amino acids, glucose, vitamins, and salts, GlutaMAXTM‐I (Gibco, Thermo Fisher Scientific, Waltham, MA, USA) (l‐glutamine), antibiotics (penicillin, streptomycin), and antifungals.

Products for electrophoresis and protein colorimetry consisted of SDS (sodium dodecyl sulfate 99% from Sigma‐Aldrich, Saint‐Louis, MO, USA) which is a detergent and a strong ionic surfactant and 2D Quant Kit (GE Healthcare, Chicago, IL, USA).

### 2.2 Cell Culture

Two culture media (30 mL) were prepared: one in which 15 TEC cylinders were immersed for 24 h (TEC medium), and another without cylinders (control medium). Gingival fibroblasts were cultured in these two media for 72 h at a temperature of 37°C in a humidified atmosphere of 5% CO_2,_ until approximately 80% confluence was reached. After this culture period, a light microscope was used to assess the overall viability of the cells. The cells were then harvested, and the proteins were extracted. Three biological replicates were included in the study.

### 2.3 Extraction and Protein Assay

The harvested cells were centrifuged several times in phosphate‐buffered saline (PBS) solution. Each pellet was solubilized in 100 µL of IEF (IsoElectric Focusing) solution (7 M urea, 2 M thiourea, 4% chaps, and 0.24% NP40), then the cells were ground on ice for 5 min and left with the IEF solution on ice for 1 h to denature the proteins. The extraction was finalized by sonication at 4°C for 30 min. The supernatant, obtained by ultracentrifugation at 150,000 × *g* for 30 min, was collected, and the proteins were assayed using the 2D Quant Kit. A reference curve was made using a solution of bovine serum albumin (BSA) of known concentration. Several tubes were prepared containing precise quantities of BSA: 0, 5, 10, 15, 20, and 30 µg. For each protein extract to be assayed, several tubes were prepared containing a precise volume of the sample. The assay was performed following the supplier's protocol. The optical density of each protein solution (BSA and protein extracts) was measured at 480 nm. Finally, the values of the results of the BSA tubes were plotted as a reference line. The resulting straight line allowed the concentration of the protein extracts to be determined.

### 2.4 Separation by Two‐Dimensional Electrophoresis

A total of six gels were prepared: three gels for the TEC and three gels as controls, to ensure good reproducibility. Two‐dimensional electrophoresis on residual fibroblast protein pellets was used to determine the optimal pH range at a predefined 12% acrylamide percentage to achieve suitable separation and fairly complete visualization of the proteins as a function of the sample. The two pH ranges tested were 4–7 and 3–10. The absence of protein below pH 4 and above pH 7 allowed the use of a pH 4–7 strip over 18 cm, making the protein separation more pronounced. A 13% acrylamide gel was preferred, which allowed easier reading detection of low molecular weight proteins. For the first dimension, 100 µg of proteins were deposited on an 18 cm immobilized pH gradient (IPG) pH 4–7 strip in the presence of IEF solution, dithiothreitol (DTT), ampholytes 4–7, and a few grains of bromophenol blue. The strip was left to rehydrate for 8 h at room temperature, covered with mineral oil. When the entire protein sample was absorbed by the strip, the latter was placed in a generator. The proteins were migrated under an electric current overnight according to a migration program based on the pH gradient, the length of the strip, and the amount of protein deposited. Migration wicks (small paper squares), moistened with 20 µL of pure water, were inserted between the generator electrode and the gel strip at its two ends: (+) and (−) pole of the generator. These wicks allow IEF migration to remove saline particles from the sample that may interfere with protein separation. For the second dimension, a 13% acrylamide gel was made. The proteins from the first dimension IPG strips were re‐equilibrated with SDS in two steps: the strip was incubated for 30 min in an equilibration solution (50 mM Tris/HCL pH 8.8, 6 M urea, 30% glycerol, 2% SDS) supplemented with 0.5% DTT, followed by incubation of the strip for 20 min in the dark in equilibration solution supplemented with 1.6% iodoacetamide. On the second‐dimension gel, 2 mL of 2% agarose liquefied at 90°C was poured. The first‐dimension strip was dipped into the agarose and positioned on the second‐dimension gel (acid side of the strip facing left). Protein markers of known molecular weights were positioned to the left of the strip. Once the agarose solidified, the gels were placed in the SDS‐PAGE migration tank containing a preprepared migration solution (25 mM Tris, 192 mM glycine, 2% SDS). Migration was performed at 10°C, 40 V for 30 min and at 100 V, 15 mA/gel for 16 h. After migration, the gels were placed under agitation for 1 h in a protein‐binding solution (ethanol 30%, acetic acid 7%) and then overnight in commercial Coomassie blue (bioRad). After staining, the gels were scanned.

### 2.5 Image Analysis of Two‐Dimensional Electrophoresis Gels

The image processing was performed with the Delta2D software (Decodon, Greifswald, Germany). The process is based on the following principle: alignment of the images, creation of a merged image, determination of the protein spots, and analysis of the quantitative differences between each spot of the same nature between the gels. The quantitative analysis was performed on the volumes of the spots. Spots that differed significantly (*t*‐test at *p* ≤ 0.05), that is, with a ratio >1.5‐fold between the two culture conditions, were selected for further analysis.

### 2.6 Digestion and Analysis by Mass Spectrometry

Spots of interest were cut out and washed in four successive acetonitrile incubations of 25 mM ammonium bicarbonate buffer. Proteins were enzymatically digested at 37°C according to the following protocol: dehydration of the gel with 100 µL acetonitrile (incubation for 10 min under agitation), removal of the acetonitrile supernatant, and addition of 20 µL trypsin solution (12 µg/mL 25 mM ammonium bicarbonate). The peptide pellet was then placed in an extraction solution (60% acetonitrile and 5% formic acid), for 5 min under agitation. Finally, the obtained peptides were analyzed by electrospray (ESI)—Quatripole‐Time‐of‐Flight (Q‐TOF) mass spectrometry (Impact HD, Bruker, Billerica, MA, USA), coupled upstream with nanoliquid chromatography (nanoLC, 300 nL/min) using a C18 reversed‐phase nanocolumn (15 cm long, 75 µm internal diameter). This column separates peptides from the complex sample according to their hydrophobicity on a 60 min increasing gradient of acetonitrile. Each peptide eluted from the column was analyzed simultaneously by the mass spectrometer in MS mode (*m*/*z* of the whole peptide) and in MSMS mode (*m*/*z* of the peptide fragments after its collision with nitrogen). The mass spectra obtained in MS/MS mode were used to identify each peptide by its amino acid sequence by querying the SwissProt protein database (https://www.expasy.org/) via the MASCOT search engine (https://www.matrixscience.com/) (2021_02; 564,638 sequences; 203,519,613 residues). The search parameters used for MASCOT were peptide mass tolerance of ±20 ppm and fragment mass tolerance of ±0.1 Da. The UniProt database (https://www.uniprot.org/) provides information about the proteins’ functions and structure. STRING database exposes known and predicted proteins’ interaction (https://string‐db.org/). The Gene Ontology (GO) knowledgebase confirmed the GO relationships between proteins (http://geneontology.org/) using the PANTHER knowledgebase (https://pantherdb.org/) [15, 16, 17, 18].

## 3 Results

### 3.1 Effects of TEC‐Released Products on the Cell Proteome

**FIGURE 2:**
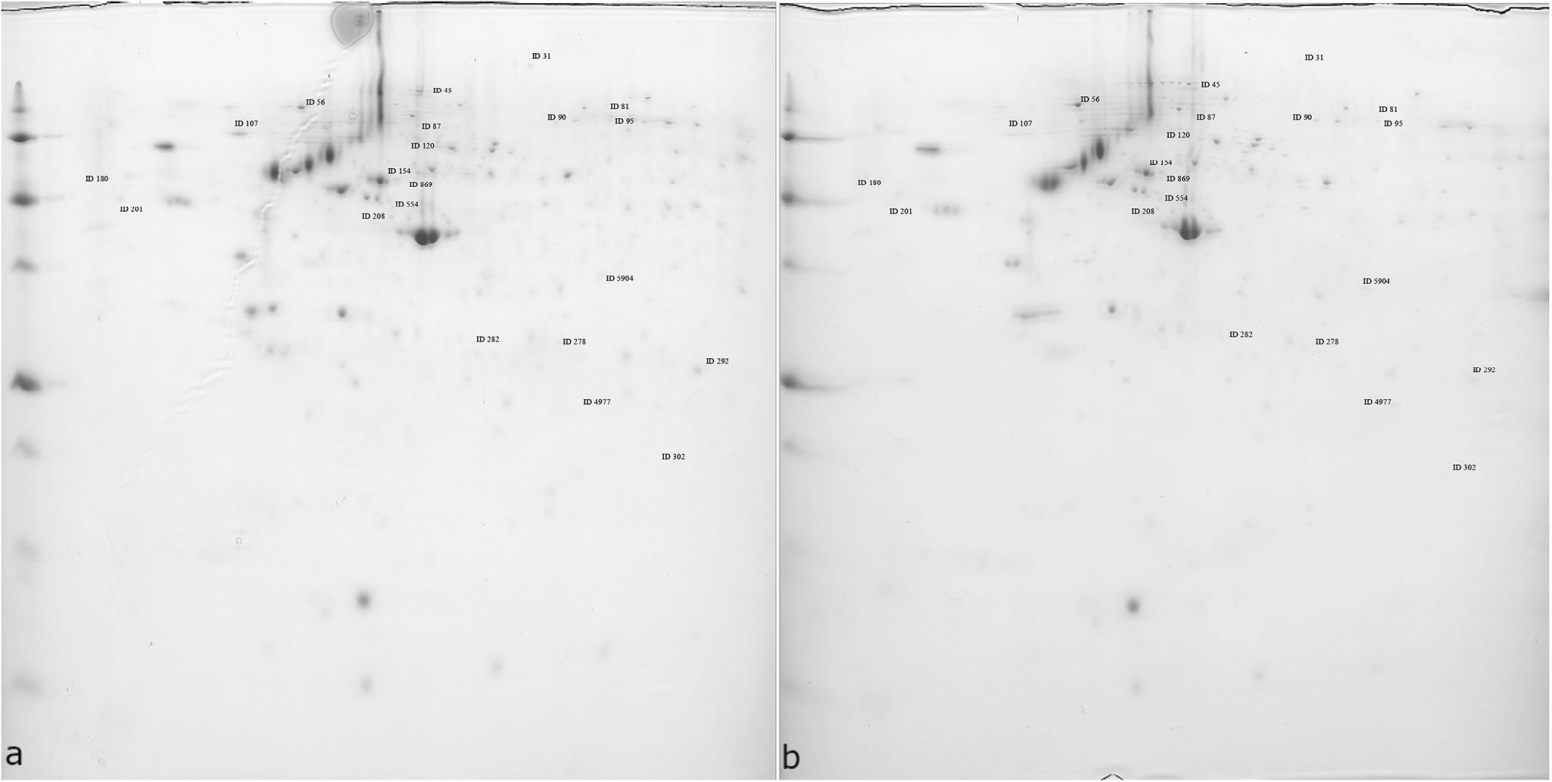
Two gels resulting from the two‐dimensional electrophoresis ((a) control medium, (b) Tetric Evo‐Ceram [TEC] medium).

The protein assay represented 3.32 µg/µL for the control and 4.04 µg/µL for the TEC medium. The six gels obtained from the dimensional electrophoresis of the two conditions could be used for analysis due to their reproducibility (Figure 2 and Appendix 1).

**FIGURE 3:**
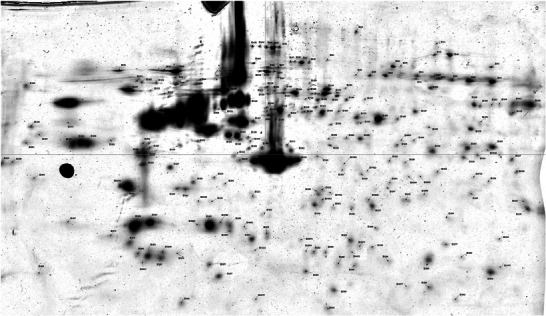
Analysis of a gel with Delta 2D.

**FIGURE 4:**
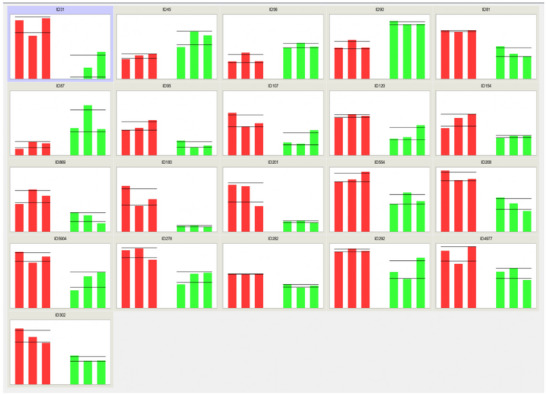
Distribution of spot volume values for each spot from the six gels (red bars: spot volume in the three control medium gels, green bars: spot volume in the three Tetric Evo‐Ceram [TEC] medium gels).

Delta2D software allowed the detection of 21 different protein spots (Figures 3 and 4), MASCOT identified 28 proteins (Table 1 and Appendix 2). Indeed, some spots contain several proteins because of the limits of separation by two‐dimensional electrophoresis. Five proteins from four spots were upregulated: actin, cytoplasmic 1 (ACTB); actin, cytoplasmic 2 (ACTG1); F‐actin‐capping protein subunit alpha‐2 (CAPZA2); endoplasmic reticulum chaperone BiP (HSPA5); and Endoplasmin (HSP90B1). Twenty‐three proteins from 17 spots were downregulated: peroxiredoxin‐6 (PRDX6), chloride intracellular channel protein 4 (CLIC4), BAG family molecular chaperone regulator 2 (BAG2), 26S proteasome regulatory subunit 6A (PSMC3), Zyxin (ZYX), MICOS complex subunit MIC60 (APOO), proteasome activator complex subunit 1 (PSME1), peptidyl‐prolyl *cis*–*trans* isomerase (FKBP10), LIM and SH3 domain protein 1 (LASP1), serine/threonine‐protein phosphatase PP1‐alpha catalytic subunit (PPP1CA), transaldolase (TALDO1), 60 kDa heat shock protein, mitochondrial (HSPD1), Parkinson disease protein 7 (PARK7), perilipin‐3 (PLIN3), endoplasmic reticulum chaperone BiP (HSPA5), pyruvate kinase (PKM), MICOS complex subunit MIC60 (APOO), Zyxin (ZYX), caldesmon (CALD1), Y‐box‐binding protein 1 (YBX1), endoplasmin (HSP90B1), collagen alpha‐1 (I) chain (COL1A1), and caveolae‐associated protein 1 (CAVIN1). Only the most abundant protein from each spot was considered responsible for the significant difference in the spot (Appendix 3). The same protein could be present in different spots, due to PTMs which explains why HSP90B, HSPA5, ZYX, and APOO are noted twice. In fact, most proteins undergo some forms of modification following translation, such as proteolytic cleavage or the addition of a modifying group to one amino acid. These modifications result in p*I* and/or molecular weight changes, thus in motility change on the 2D electrophoresis gel.

**TABLE 1:** Main role of the identified proteins.

Regulation	Protein	Main role
+	Actin, cytoplasmic 1	Cellular architecture
+	Actin, cytoplasmic 2	Cellular architecture
+	F‐actin‐capping protein subunit alpha‐2	Cellular architecture
+	Endoplasmin	Structural modification of proteins
−	Peroxiredoxin‐6	Hydrogen peroxide reduction Phospholipase activity
−	Chloride intracellular channel protein 4	Trans‐epithelial transport Maintenance of intracellular pH Regulation of cell volume Cell membrane stabilization
−	BAG family molecular chaperone regulator 2	Binding to Hsc70/Hsp70 (involved in protein quality control)
−	26S proteasome regulatory subunit 6A	Maintenance of cell proteome homeostasis by degradation of misfolded or damaged proteins
−	Zyxin	Messenger involved in cell adhesion to the extracellular matrix
−	Proteasome activator complex subunit 1	Treatment of MHC class I peptides (presentation of intracellular proteins to cytotoxic T cells)
−	Peptidyl‐prolyl cis‐trans isomerase FKBP10	Acceleration of protein folding during protein synthesis
−	LIM and SH3 domain protein 1	Actin binding, organization of the cytoskeleton
−	60 kDa heat shock protein, mitochondrial	Protein folding chaperone and peptide assembly
−	Parkinson disease protein 7	Role in cell protection (against oxidative stress and cell death)
−	Perilipin‐3	Transport of mannose 6‐phosphate receptors (MPR) from endosomes to the trans‐Golgi network
−	Endoplasmic reticulum chaperone BiP	Maintenance of newly synthesized proteins in a state competent for folding
−	Pyruvate kinase PKM	Aerobic respiration (glycolytic path)
−	MICOS complex subunit MIC60	Maintenance of crista junctions, inner membrane architecture, mitochondrial ridge morphology
−	Y‐box‐binding protein 1	RNA stabilization, mRNA splicing, DNA repair, transcription regulation
−	Endoplasmin	Structural modification of proteins
−	Caveolae‐associated protein 1	Formation and organization of caveolae (plasma membrane invagination, receptor for certain hormones and cytokines)

Spot numbers 90, 81, 5904, 95, and 31 contained several proteins, respectively, CAPZA2 with HSPA5, ZYX with APOO, LASP1 with PPP1CA and TALDO1, APOO with ZYX and CALD1, and HSP90B1 with COL1A1.

### 3.2 Biological Significance of the Regulated Proteins

**FIGURE 5:**
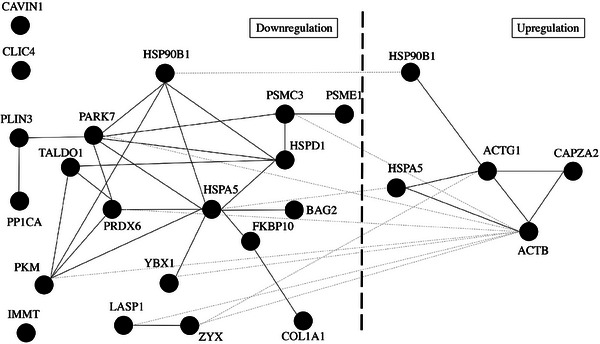
Proteins interactions from the STRING database (black solid lines: the link between proteins from the same regulation, gray dotted lines: the link between proteins from different regulations).

The UniProt database showed that all these proteins were involved in cellular architecture, structural modifications and quality control of proteins, cellular homeostasis, and metabolic pathways. The STRING database revealed the interactions between the regulated proteins (Figure 5). The GO enrichment analysis showed that four pathways were significantly affected. The most affected biological processes were: cellular response to chemical stimulus and cellular response to organic substance (Table 2). The protein–protein interaction (PPI) enrichment *p* value was 2.26e‐06, which indicates the regulated proteins had more interactions among themselves than expected for a similarly sized random set of proteins. The PPI enrichment *p* value further suggests that these proteins were at least partially biologically connected as a group. The GO knowledgebase and PANTHER knowledgebases confirmed these results.

**TABLE 2:** Gene Ontology (GO) enrichment analysis of biological processes that were affected.

GO‐term	Description	CIN	Expected	*p*	Proteins
GO:0032091	Negative regulation of protein binding	4 of 93	0.10	3.81e‐06	PP1CA, **ACTB**, PARK7, BAG2
GO:0006457	Protein folding	5 of 220	0.25	4.25e‐06	HSPA5, HSPD1, **HSP90B1**, FKBP10, BAG2
GO:0071310	Cellular response to organic substance	10 of 1777	1.99	9.32e‐06	YBX1, HSPA5, COL1A1, HSPD1, **HSP90B1**, ZYX, **ACTB**, PARK7, PKM, **ACTG1**
GO:0070887	Cellular response to chemical stimulus	12 of 2397	2.68	2.47‐06	YBX1, HSPA5, COL1A1, HSPD1, **HSP90B1**, ZYX, **ACTB**, PARK7, PKM, **ACTG1**, PRDX6, CLIC4

*Note:* Bold, upregulated; underlined, downregulated. Count In Network (CIN): first number (proteins in the network) and second number (proteins in the network and in the background). Expected: number of genes you would expect in your list for this category, based on the reference list. *p*: raw *p* value as determined by Fisher's exact test or binomial statistic.

## 4 Discussion

To our knowledge, this is the first study assessing the side effects of the release of a resin composite on human gingival fibroblasts by a proteomic approach.

The proteome is a complex, consisting of several hundred different proteins, and a highly dynamic system due to quantitative variations in proteins [19]. Its analysis (qualitative and quantitative) requires the development of suitable tools capable of separating, analyzing, and identifying it [12]. The techniques used are evolving very rapidly to answer increasingly precise biological questions. The combination of a 2D electrophoresis gel to separate proteins, followed by their identification by mass spectrometry, is the reference method, relatively easy to implement, reliable, and sufficient for an initial proteomic approach to the subject.

Quantitative changes in proteome were observed when the cells were immersed in the TEC medium. Thus, resin composite released products caused early adverse effects on cells, which could not be detected by a cytotoxicity test as the MTT test, which only assesses the cell viability. These products were probably monomers such as Bis‐GMA, UDMA or Bis‐EMA, or other monomers not specified in the MSDS or other products from the TEC composition. The proteomic approach allowed precise and early assessment of cytotoxicity.

Three proteins were upregulated. ACTB and ACTG1 play a key role in cell motility [20] and DNA damage repair (for ACTB) [21]. CAPZA2 is a capping protein that binds to the fast‐growing ends of actin filaments, blocking subunit exchange at these ends. In a gastric cancer study, it was shown to regulate apoptosis or cell cycle progression [22].

Sixteen proteins were downregulated. PRDX6 is a member of the peroxiredoxins family (six enzymes) whose role is to prevent cellular oxidative stress induced by ROS. PRDX6 might be involved in regulating cell proliferation and apoptosis for example. A study demonstrated that the knockdown of PRDX6 could induce higher levels of ROS, and this could activate the JAK1/STAT1 signaling pathway, which is related to cell proliferation, apoptosis, differentiation, and inflammatory response [23]. ZYX could be a signaling protein (between the cytoskeleton and the nucleus) regulating cell growth and/or differentiation, be involved in actin filament stimulation, and play a role in the spatial control of these filaments. In one study, reduced ZYX expression had an impact on cell propagation and proliferation [24]. PSME1 is thought to be involved in immunoregulation. A number of studies suggested that its reduction is consistent with cancer initiation and progression due to impaired immunoprotection [25]. FKBP10 inactivation could lead to reduced cell proliferation [26]. Inhibition of COL1A1 is thought to reduce cell proliferation [27]. Lack of PKM expression would inhibit cell proliferation and lead to apoptosis [28].

One protein was upregulated in one spot and downregulated in another. Encoded HSP90B1 is localized in melanosomes and the endoplasmic reticulum. Its expression is associated with a variety of pathogenic conditions, including tumor formation [29].

The identity of these proteins reveals that the products released by the resin composite lead to a disruption of cellular function (decrease in certain proteins involved in DNA replication, such as YBX1, for example [30], and increase in cytoskeleton components such as actin). Thus, the products rejected by the resin composite would impact proteins essential to the vitality of the cell.

The STRING database revealed the different affected reactome pathways by predicting protein‐protein interactions. Among them, the most concerned was ATF6‐mediated unfolded protein response. This pathway activates the ATF6 in response to endoplasmic reticulum stress to regulate the cell to maintain folding capacity. By analyzing the GO biological process nonsignificatively reported with the proteins, the negative regulation of TRAIL‐activated apoptotic signaling pathway (GO:1903122) with their parent terms (negative regulation of extrinsic apoptotic signaling pathway via death domain receptors GO:1902042 PARK7, regulation of extrinsic apoptotic signaling pathway via death domain receptors GO:1902041 PARK7, regulation of extrinsic apoptotic signaling pathway GO:2001236 PARK7, regulation of apoptotic signaling pathway GO:2001233 PARK7, regulation of apoptotic process GO:0042981 HSPA5‐HSPD1‐HSP90B1‐ACTB‐PARK7, and regulation of programmed cell death GO:0043067 HSPA5‐HSPD1‐HSP90B1‐ACTB‐PARK7). The fold enrichment of these GO biological processes is over 1 (indicating the category is overrepresented in the experiment) with a raw *p* value under 0.05 (probability that the number of genes observed occurred randomly) but with a false discovery rate (FDR) over 0.05. PARK7 is an antioxidant protein that protects cells against oxidative stress and cell death [31].

Furthermore, PTMs were identified. Indeed, a considerable number of newly translated proteins undergo changes through PTMs to become other proteins implying other cellular functionality. The different forms of a protein are called proteoforms [32]. Potential modifications encompass the elimination of N‐terminal amino acid residues and covalent modifications (acetylation, phosphorylation, methylation, ubiquitinylation, and glycosylation). A single amino acid sequence, encoded by specific genes, can give rise to a multitude of proteoforms through the action of over 300 PTMs. These modifications occur after protein synthesis and over time. This phenomenon is attributable to interactions with the fluctuating biochemical milieu [33, 34]. PTMs represent an important mechanism for regulating protein function: enzymatic activity, protein interactions, and subcellular localization. Further studies should determine the nature of the identified PTMs to provide a more reliable picture of how these signaling proteins integrate and transmit information within the cell after exposition of the monomers release and to give more information on the potential damages caused.

For example, endoplasmin, which is the most abundant protein in spots 56 and 31, is overexpressed in spot 56 and underexpressed in spot 31 compared to control groups. The MASCOT search indicates oxidation, carbamidomethylation, and phosphorylation for the proteins from spot 56, whereas it indicates oxidation, acetylation, and carbamidomethylation for the proteins from spot 31. PTMs are therefore responsible for this difference.

Finally, further studies evaluating the effects of different monomers and other products from resin composites on the various cellular proteomes would be relevant. They would help identify the most deleterious components. Nilsen et al. already assessed the effect of TEGDMA on monocytes’ proteome [35]. They reported changes in protein regulation and biological pathway, but different to ours. Moreover, the combination of different products can also have a “cocktail effect”, hence the interest of this study, which reflects the effect of a product used clinically.

### 4.1 Limits

The use of cylinders is an approximation of the clinical reality, where the surface area of the resin composite in contact with fibroblasts is generally smaller. It should also be emphasized that the products released cannot stagnate 24 h in the oral cavity due to the gingival turnover and regular salivary renewal.

Two‐dimensional electrophoresis and Coomassie blue staining pose limitations in the detection of protein numbers. In addition, taking into account the most abundant protein of the spot of interest is potentially a noninterpretation of other underlying protein phenomena.

Even with all the information provided by proteomics, it is difficult to know at what stage the cells are between a healthy and a dead cell, after TEC exposition. Besides, we do not know what mechanism leads to cell death: apoptosis, autophagy, necrosis, or others.

## 5 Conclusion

This experimental study proposed a proteomic approach to analyze the potential toxicity of a resin composite on human gingival fibroblasts. It showed that the products released have led to changes in the fibroblast proteome. Thus, under the conditions of this study, the resin composite released products can cause early adverse effects on cells, but without ceasing to carry out their functions. Further studies are needed to ascertain the maximum dose of products released allowing bio‐inertia, that is, without proteomic modifications and what volume of resin composite it corresponds to; then, to identify which monomer(s) and/or other products of the resin composite are involved in these protein modifications, and to what extent.

## Author Contributions

**Yohann Flottes:** contributed to experimentations, data acquisition, analysis and interpretation, drafted the manuscript. **Elisabeth Dursun:** contributed to conception and design, data interpretation, drafted and critically revised the manuscript. All authors gave their final approval and agree to be accountable for all aspects of the work.

## Conflicts of Interest

The authors declare no conflicts of interest.

## Data Availability

Data available in article Supporting Information.

## Appendix 2

### 2.1

Spots identified as significantly different between control and condition gels

**Table:**

	Control gels			TEC gels					
Spot	Average volumes	RSD	n	Average volumes	RSD	*n*	Ratio TEC/control	Ratio Control/TEC	*p* (*t*‐test)
87	30.60523	28.84579	3	96.58562	25.21531	3	3.16	*0.31*	0.02272
45	43.9451	7.16638	3	76.71242	5.00858	3	1.75	*0.57*	0.00074
90	23.07974	15.61129	3	37.42614	15.87582	3	1.62	*0.61*	0.04325
56	398.20523	24.47838	3	629.95556	9.13291	3	1.58	*0.63*	0.04431
292	224.04183	8.25704	3	147.26275	23.01288	3	*0.66*	1.51	0.0482
282	66.16471	7.08899	3	42.80261	18.57443	3	*0.65*	1.53	0.02318
4977	20.96078	10.57759	3	13.34248	20.26279	3	*0.64*	1.56	0.03688
554	36.57255	1.77378	3	21.35294	8.97282	3	*0.58*	1.72	0.00044
81	139.23399	6.65279	3	78.03268	29.68953	3	*0.56*	1.78	0.02561
278	48.65882	15.75984	3	26.17778	6.20034	3	*0.54*	1.85	0.0154
120	76.34771	8.17797	3	40.46405	23.03247	3	*0.53*	1.88	0.01063
5904	24.7817	11.45516	3	13.1268	17.93511	3	*0.53*	1.88	0.01108
154	67.68758	13.26251	3	35.58431	6.98572	3	*0.53*	1.88	0.0082
302	37.85621	18.25287	3	19.76993	23.22742	3	*0.52*	1.92	0.03683
208	53.69281	12.71471	3	27.06405	31.63077	3	*0.50*	2.00	0.02631
107	670.49412	20.84191	3	309.99085	32.66334	3	*0.46*	2.17	0.04179
869	102.56863	18.84325	3	42.78693	40.15337	3	*0.42*	2.38	0.03081
95	56.19739	6.73518	3	21.24575	39.40497	3	*0.38*	2.63	0.00577
201	77.66144	30.27817	3	19.06013	15.39336	3	*0.25*	4.00	0.02496
31	52.19869	12.51105	3	11.15948	87.0909	3	*0.21*	4.76	0.00772
180	101.52157	27.25926	3	15.65882	18.86414	3	*0.15*	6.66	0.01203

## Appendix 3

### 3.1

List of proteins upregulated and downregulated in fibroblast cells exposed to TEC.

**Table:**

Spot	Ratio	Variation	*p* (*t*‐test)	Protein name	Mascot score	Peptides number	Swiss‐Prot number
87	3.16	Upregulation	0.02272	**Actin, cytoplasmic 1**	1471	45	P60709
45	1.75	Upregulation	0.00074	**Actin, cytoplasmic 2**	1657	50	P63261
90	1.62	Upregulation	0.04325	**F‐actin‐capping protein subunit alpha‐2**	200	5	P47755
				Endoplasmic reticulum chaperone BiP	151	4	P11021
56	1.58	Upregulation	0.04431	**Endoplasmin**	11,690	339	P14625
292	1.51	Downregulation	0.0482	**Peroxiredoxin‐6**	717	29	P30041
282	1.53	Downregulation	0.02318	**Chloride intracellular channel protein 4**	224	10	Q9Y696
4977	1.56	Downregulation	0.03688	**BAG family molecular chaperone regulator 2**	129	4	O95816
554	1.72	Downregulation	0.00044	**26S proteasome regulatory subunit 6A**	100	5	P17980
81	1.78	Downregulation	0.02561	**Zyxin**	345	18	Q15942
				MICOS complex subunit MIC60	288	9	Q16891
278	1.85	Downregulation	0.0154	**Proteasome activator complex subunit 1**	206	8	Q06323
120	1.88	Downregulation	0.01063	**Peptidyl‐prolyl cis‐trans isomerase FKBP10**	908	35	Q96AY3
5904	1.88	Downregulation	0.01108	**LIM and SH3 domain protein 1**	964	37	Q14847
				Serine/threonine‐protein phosphatase PP1‐alpha catalytic subunit	280	7	P62136
				Transaldolase	169	8	P37837
154	1.88	Downregulation	0.0082	**60 kDa heat shock protein, mitochondrial**	836	22	P10809
302	1.92	Downregulation	0.03683	**Parkinson disease protein 7**	1672	59	Q99497
208	2.00	Downregulation	0.02631	**Perilipin‐3**	1479	46	O60664
107	2.17	Downregulation	0.04179	**Endoplasmic reticulum chaperone BiP**	16,046	387	P11021
869	2.38	Downregulation	0.03081	**Pyruvate kinase PKM**	244	10	P14618
95	2.63	Downregulation	0.00577	**MICOS complex subunit MIC60**	541	25	Q16891
				Zyxin	285	17	Q15942
				Caldesmon	188	10	Q05682
201	4.00	Downregulation	0.02496	**Y‐box‐binding protein 1**	661	15	P67809
31	4.76	Downregulation	0.00772	**Endoplasmin**	233	6	P14625
				Collagen alpha‐1 (I) chain	138	5	P02452
180	6.66	Downregulation	0.01203	**Caveolae‐associated protein 1**	540	28	Q6NZI2

*Note:* The proteins considered responsible of the significant difference of the spot are in bold characters.
